# Supplementary material for: The Role of Multimodal Imaging in Pathological Response Prediction of Locally Advanced Cervical Cancer Patients Treated by Chemoradiation Therapy Followed by Radical Surgery
Source: Cancers (Basel). 2023 Jun 6;15(12):3071. doi: 10.3390/cancers15123071 (PMC10296350; doi:10.3390/cancers15123071)
Supplement: Supplementary file 1 [file cancers-15-03071-s001.zip › cancers-2317157-supplementary.pdf]

Supplementary table S1. Ultrasound, MRI and PET/CT parameters that significantly differed between patients with partial response and those with complete response at "Baseline", "early", "final" examinations, and at the analysis of the changes between the quantitative variables of two longitudinal examinations in terms of percentage variation (Δ "baseline"-"early" examinations and Δ "baseline"-"final" examinations)

Characteristic	Partial response n=48	Complete response n=40	p value
**"Baseline" examination**			
US			
Color score 4, n (%)	33 (68.8)	19 (47.5)	**0.013**
VI	39.5 (13.0-69.0)	46.5 (14.0-86.0)	**0.037**
VFI	15.0 (4.0-31.0)	18 (5.0-49.0)	**0.036**
Tumor peak enhancement, a.u. (n=86)	18150.9 (1100.0-84580.5)	28024.7 (1041.7-70106.0)	**0.0006**
Rise time, s (n=86)	8.7 (4.7-19.2)	7.6 (4.4-22.7)	**0.038**
Wash in rate (n=86)	2958.5 (240.5-160506.3)	5329.1 (85.6-23037.6)	**0.003**
MRI			
None characteristic included in the present study			
PET/CT			
SUV_{max}	12.7 (4.0-35.0)	17.3 (7.4-37.2)	**0.003**
SUV_{mean}	7.6 (2.8-23.2)	10.2 (4.3-23.7)	**0.001**
**"Early" examination**			
US (n=74)			
Maximum tumor diameter, mm	45.0 (19.0-75.0)	36.0 (13.0-61.0)	**0.006**
Tumor volume, cm^3	26.5 (2.0-107.9)	14.3 (1.1-81.8)	**0.019**
VI (n=74)			**0.024**
MRI			
Maximum tumor diameter, mm	13.4 (0.3-101.3)	5.6 (0.1-57.2)	**0.003**
Tumor volume, cm^3	35.0 (10.0-70.0)	28.5 (10-0-62.0)	**0.001**
PET/CT			
MTV	89.9 (4.5-779.4)	32.2 (4.2-1206.4)	**0.004**
TLG	17.7 (1.3-106.5)	8.5 (1.6-94.6)	**0.003**
**"Final" examination**			
US			
None characteristic included in the present study			
MRI (n=82)			
Maximum tumor diameter, mm	0.5 (0-23.6)	0.1 (0-2.4)	**<0.0001**
Tumor volume, cm^3	12 (0-58)	8.5 (0-20)	**0.001**
Evaluation according to high DWI SI plus $ADC_{mean} \leq 1.1 \times 10^{-3}$ mm^2/s (n=82), n (%)	28/44 (63.6)	7/38 (18.4)	**< 0.0001**
PET/CT			
SUV_{max}	2.7 (1.2-10.5)	2.3 (1.3-4.4)	**0.001**
SUV_{mean}	2.1 (1.1-5.7)	1.8 (1.1-2.7)	**0.004**
TLG	4.8 (0.7-138)	3.2 (0.1-25.7)	**0.020**
**Δ "baseline"-"early" examination**			
US			
Δ Maximum tumor diameter %	8.7 (−41.7-50.0)	20.8 (−13.2 -71.1)	**0.012**
Δ Tumor volume %	16.4 (−231.0-90.0)	55.2 (−37.5-95.2)	**0.017**
Δ Tumor peak enhancement %	−56.5 (−2927.4-62.0)	−1 (−676.1-77.1)	**0.031**
Δ Wash in rate %	−68.9 (−1588.8-95.3)	1.6 (−1101.2-89.4)	**0.015**
MRI			
Δ Maximum tumor diameter %			
Δ Tumor volume %			
PET/CT			
Δ SUV_{max} %	36.3 (-11.3-84)	52.1 (-9.1-84.9)	**< 0.0001**
Δ SUV_{mean} %	33 (-21.6-81.9)	52.3 (-8.7-86.5)	**< 0.0001**
Δ MTV %	37.8 (-254.2-93.8)	70 (-140.1-97.2)	**0.001**

Δ TLG %	59.8 (-141-96.6)	84.3 (-80.7-99.4)	**0.0001**
**Δ "baseline"-"final" examination**			
US			
Evaluation not performed			
MRI			
Δ Maximum tumor diameter %	98.3 (19.5-100.0)	99.6 (94.3-100.0)	**0.001**
Δ Tumor volume %	72.1 (10.8-100.0)	80.5 (56.5-100.0)	**0.001**
Δ ADC_{mean} % (n=82)	-15.3 (-64.7-10)	-33.3 (-157.1-14.3)	**0.007**
PET/CT			
Δ SUV_{max} %	77.4 (26.3-91.8)	87 (67-93.1)	**< 0.0001**
Δ SUV_{mean} %	73.7 (12.7-91.5)	83.1 (63.6-91.8)	**< 0.0001**
Δ TLG %	97.6 (12.8-99.8)	98.8 (66.3-100)	**0.003**

Results are presented as median (min-max), except where indicated. P value was calculated with two sided Mann-Whitney U test or Pearson's Chi Square test as appropriate. Bold font highlights statistically significant value. US: Ultrasound. MRI: Magnetic Resonance imaging. PET/CT: Positron Emission Tomography/Computer Tomography. VI: Vascularization Index. VFI: Vascularization Flow Index. SUV: Standardized Uptake Value. MTV: Metabolic Tumor Volume. TLG: Total Lesion Glycolysis. DWI SI: Diffusion Weighted Imaging Signal Intensity. ADC: Apparent Diffusion Coefficient.
